# Supplementary material for: Protective Effect of Betulin on Streptozotocin–Nicotinamide-Induced Diabetes in Female Rats
Source: Int J Mol Sci. 2024 Feb 10;25(4):2166. doi: 10.3390/ijms25042166 (PMC10888537; doi:10.3390/ijms25042166)
Supplement: Supplementary file 1 [file ijms-25-02166-s001.zip › ijms-2837121-supplementary.pdf]

Supplementary Materials

Article

Protective Effect of Betulin on Streptozotocin–Nicotinamide-Induced Diabetes in Female Rats

Feyisayo O. Adepoju [1], Ksenia V. Sokolova [1,2], Irina F. Gette [1,2], Irina G. Danilova [1,2], Mikhail V. Tsurkan [3], Alicia C. Mondragon [4], Elena G. Kovaleva [1,*] and Jose Manuel Miranda [4,*]

[1] Department of Technology for Organic Synthesis, Institute of Chemical Technology, Ural Federal University, Mira 19, 620002 Yekaterinburg, Russia; besee010@gmail.com (F.O.A.); xenia.socolova@gmail.com (K.V.S.); i.goette@yandex.ru (I.F.G.); ig-danilova@yandex.ru (I.G.D.)
[2] Institute of Immunology and Physiology, Russian Academy of Sciences, 620049 Yekaterinburg, Russia
[3] Leibniz Institute of Polymer Research, 01069 Dresden, Germany; tsurkan@ipfdd.de
[4] Departamento de Química Analítica, Nutrición y Bromatología, Campus Terra, Universidade da Santiago de Compostela, 27002 Lugo, Spain; alicia.mondragon@usc.es
* Correspondence: e.g.kovaleva@urfu.ru (E.G.K.); josemanuel.miranda@usc.es (J.M.M.)

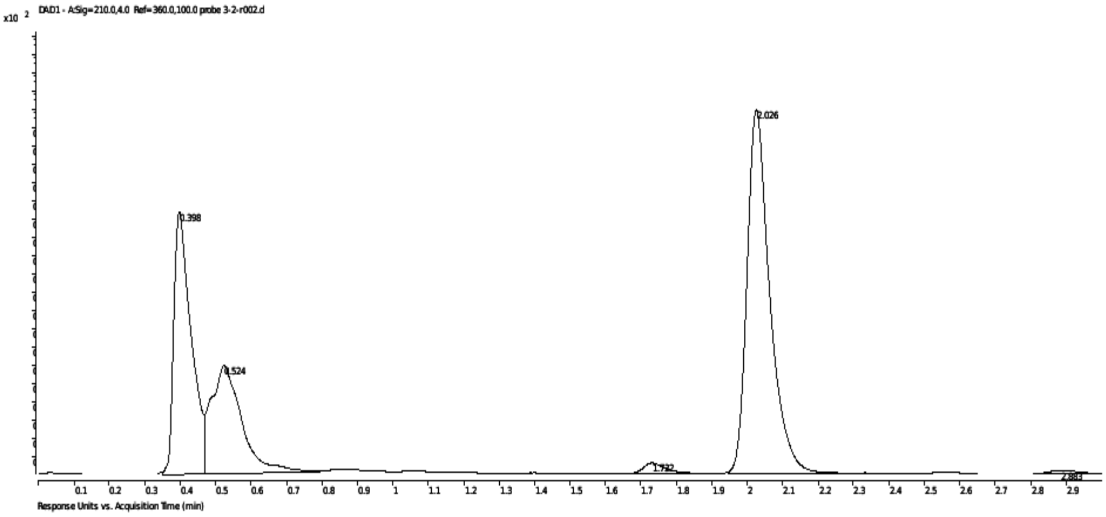

Figure S1. HPLC chromatogram of betulin at a retention time of 2.026 minutes.

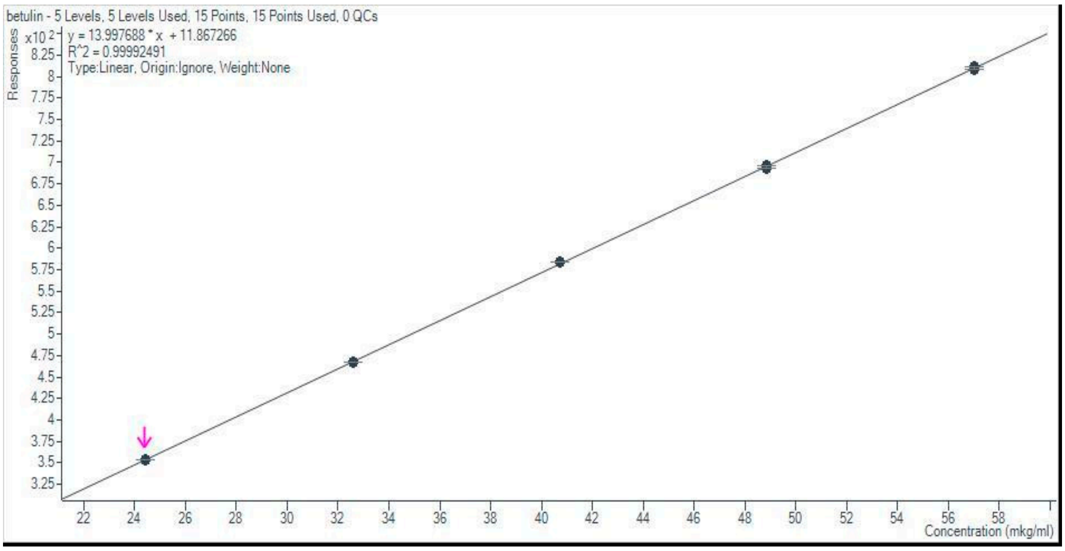

Figure S2. Calibration curve of betulin standard at different concentrations.

Table S1. Concentration of betulin emulsion at different time points before, during, and after the stability tests.

Samples	Exposure time at 55 °C, in hours	Mass concentration of betulin in emulsion, g/dm^3	Emulsion layering, yes/no
Emulsion	0	4.12 ± 0.11	no
Vial 1	180	4.06 ± 0.04	no
Vial 2	360	4.07 ± 0.07	no
Vial 3	540	4.09 ± 0.11	no
Vial 4	720	4.17 ± 0.08	no

Table S2. Effect of betulin intervention on oxidative stress parameters. Data are presented as mean ± standard error of mean (SEM); INT–healthy rats without any treatment, NC—healthy rats administered water, NC + BE50—healthy rats administered betulin (50 mg/kg), T2D—diabetic rats administered water, T2D + BE20—Diabetic rats administered betulin (20 mg/kg), T2D + BE50—diabetic rats administered betulin (50 mg/kg).

	Glutathione in blood plasma, µmol/L	MDA in blood plasma µmol/L	SOD units/ GmHb	CAT mmol/GmHb
INT	20.5 ± 5.3	3.19 ± 0.23	112.5 ± 5.0	50.2 ± 2.0
NC	88.2 ± 8.4	3.75 ± 0.38	132.4 ± 3.5	48.5 ± 2.8
NC + BE50	47.2 ± 3.4	2.60 ± 0.31	130.8 ± 6.6	37.0 ± 2.6
T2D	30.9 ± 4.8	6.27 ± 0.25	132.8 ± 8.7	48.9 ± 1.9
T2D + BE20	77.7 ± 7.9	5.73 ± 0.43	132.2 ± 4.6	43.3 ± 1.9
T2D + BE50	46.1 ± 5.3	4.46 ± 0.19	110.9 ± 13.7	45.3 ± 1.2

Table S3. Effect of betulin on oral glucose tolerance (OGTT) and Area under the curve (AUC) in rats. Data are presented as mean ± standard error of mean (SEM); INT—healthy rats without any treatment, T2D—diabetic rats administered water, T2D + BE20—Diabetic rats administered betulin (20 mg/kg), T2D + BE50—diabetic rats administered betulin (50 mg/kg).

	Glucose level, mmol/L				AUC, mmol/L·min
	Before load	30 minutes	60 minutes	120 minutes	
INT	5.9 ± 0.3	8.8 ± 0.3	6.5 ± 0.8	5.3 ± 0.8	802 ± 35
T2D	10.3 ± 0.4	15.7 ± 1.9	14.2 ± 0.7	12.4 ± 0.8	1636 ± 113
T2D + BE20	6.3 ± 0.6	8.1 ± 1.0	6.4 ± 0.5	4.6 ± 0.4	721 ± 53
T2D + BE50	7.2 ± 0.5	10.7 ± 1.9	9.9 ± 1.7	6.6 ± 0.6	1061 ± 136

Table S4. Effect of betulin on serum insulin concentration and HOMA-IR. Data are presented as mean ± standard error of mean (SEM); INT–healthy rats without any treatment, NC + BE50—healthy rats administered betulin (50 mg/kg), T2D—diabetic rats administered water, T2D + BE20—Diabetic rats administered betulin (20 mg/kg), T2D + BE50—diabetic rats administered betulin (50 mg/kg).

	Insulin µIU/mL	HOMA-IR
INT	1.9 ± 0.25	0.5 ± 0.09
NC + BE50	2.12 ± 0.72	0.81 ± 0.28
T2D	1.98 ± 0.05	0.91 ± 0.06
T2D + BE20	1.93 ± 0.48	0.55 ± 0.19
T2D + BE50	2.22 ± 0.26	0.72 ± 0.13

Table S5. Effect of betulin intervention on hepatic and renal functions. Data are presented as mean ± standard error of mean (SEM); INT–healthy rats without any treatment, NC–healthy rats administered water, NC + BE50—healthy rats administered betulin (50 mg/kg), T2D—diabetic rats administered water, T2D + BE20—Diabetic rats administered betulin (20 mg/kg), T2D + BE50—diabetic rats administered betulin (50 mg/kg).

	AST µmol/min·L	ALT µmol/min·L	ALP µmol/min·L	Total protein g/L	Urea mmol/L	Creatinine µmol/L
INT	16.1 ± 0.6	12.6 ± 0.6	51.5 ± 3.3	70.5 ± 1.3	5.2 ± 0.2	62.3 ± 1.5
NC	18.5 ± 0.8	12.8 ± 0.7	48.5 ± 4.3	67.3 ± 2.2	5.9 ± 0.3	60.2 ± 1.7
NC + BE50	17.4 ± 1.7	13.1 ± 0.8	32.0 ± 2.4	67.6 ± 1.3	5.2 ± 0.4	64.3 ± 1.6
T2D	24.7 ± 1.7	18.6 ± 1.2	40.8 ± 4.0	64.3 ± 2.1	7.9 ± 0.4	67.7 ± 0.7
T2D + BE20	21.7 ± 1.4	13.6 ± 0.8	30.4 ± 1.4	62.1 ± 1.6	7.0 ± 0.3	68.0 ± 3.3
T2D + BE20	20.2 ± 2.6	11.3 ± 1.4	27.6 ± 1.7	68.0 ± 1.5	5.8 ± 0.1	64.3 ± 1.9

Table S6. Effect of betulin on blood glucose, glycosylated haemoglobin, α-amylase, and glucose tolerance. Data are presented as mean ± standard error of mean (SEM); INT—healthy rats without any treatment, NC—healthy rats administered water, NC + BE50—healthy rats administered betulin (50 mg/kg), T2D—diabetic rats administered water, T2D + BE20—Diabetic rats administered betulin (20 mg/kg), T2D + BE50—diabetic rats administered betulin (50 mg/kg).

	Glucose mmol/L	HbA1c %	α-amylase mg/s·L
INT	5.9 ± 0.3	4.5 ± 0.2	30.9 ± 1.8
NC	7.7 ± 0.3	4.4 ± 0.3	34.7 ± 0.7
NC + BE50	8.6 ± 0.1	4.8 ± 0.4	45.0 ± 2.3
T2D	10.3 ± 0.4	6.1 ± 0.4	43.7 ± 1.6
T2D + BE20	6.3 ± 0.6	5.5 ± 0.7	38.1 ± 1.2
T2D + BE20	7.2 ± 0.5	4.9 ± 0.2	35.1 ± 1.6

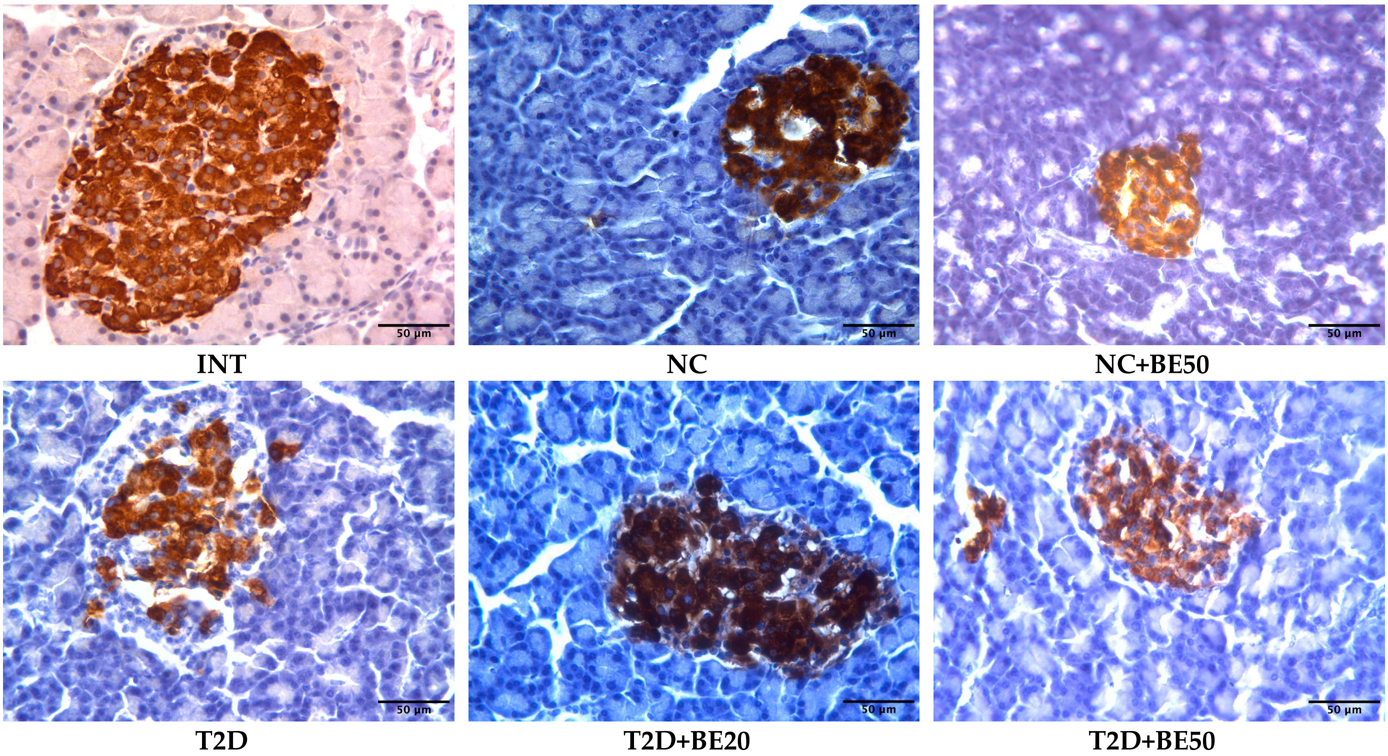

Figure S3. Pancreatic sections with immunohistochemical stain for insulin antibodies (brown) at ×400 magnification. INT—healthy rats without any treatment, NC—healthy rats administered water, NC + BE50—healthy rats administered betulin (50 mg/kg), T2D—diabetic rats administered water, T2D + BE20—Diabetic rats administered betulin (20 mg/kg), T2D + BE50—diabetic rats administered betulin (50 mg/kg).
